# Severe hypovitaminosis D correlates with increased inflammatory markers in HIV infected patients

**DOI:** 10.1186/1471-2334-13-7

**Published:** 2013-01-07

**Authors:** Thiphaine Ansemant, Sophie Mahy, Christine Piroth, Paul Ornetti, Stephanie Ewing, Jean-Claude Guilland, Delphine Croisier, Laurence Duvillard, Pascal Chavanet, Jean-Francis Maillefert, Lionel Piroth

**Affiliations:** 1 Infectious Diseases Department, CHU, Dijon, 21079, France; 2 Rheumatology Department, CHU, Dijon, F-21078, France; 3 INSERM U866 and Laboratory of biochemistry, CHU, Dijon, France; 4 University of Burgundy, Dijon, F-21079, France; 5 INSERM U887, Dijon, F-21079, France

**Keywords:** Antiretroviral therapy, Bone metabolism, HIV, Inflammation, 25-hydroxyvitamin D

## Abstract

**Background:**

Even though it has been suggested that antiretroviral therapy has an impact on severe hypovitaminosis D (SHD) in HIV infected patients, it could be speculated that the different levels of residual inflammation on HAART (Highly Active Anti Retroviral Therapy) could contribute to SHD and aggravate bone catabolism in these patients.

**Methods:**

A cross-sectional study was carried out in an unselected cohort of 263 HIV infected outpatients consulting during Spring 2010. Clinical examinations were performed and medical history, food habits, sun exposure and addictions were collected. Fasting blood samples were taken for immunological, virological, inflammation, endocrine and bone markers evaluations.

**Results:**

Ninety-five (36%) patients had SHD. In univariate analysis, a significant and positive association was found between SHD and IL6 (p = 0.001), hsCRP (p = 0.04), increased serum C-Telopeptides X (CTX) (p = 0.005) and Parathyroid Hormon (PTH) (p < 0.0001) levels. In multivariate analysis, SHD deficiency correlated significantly with increased IL-6, high serum CTX levels, lower mean daily exposure to the sun, current or past smoking, hepatitis C, and functional status (falls), but not with the time spent on the current HAART (by specific drug or overall).

**Conclusions:**

SHD is frequent and correlates with inflammation in HIV infected patients. Since SHD is also associated with falls and increased bone catabolism, it may be of interest to take into account not only the type of antiretroviral therapy but also the residual inflammation on HAART in order to assess functional and bone risks. This finding also suggests that vitamin D supplementation may be beneficial in these HIV-infected patients.

## Background

There is growing evidence that 25 OH vitamin D3 deficiency is highly prevalent in HIV-infected patients, and induces a number of clinical consequences [1-4]. Low serum 25-OHD3 concentrations induce bone disorders like osteomalacia, are associated with increased bone resorption due to secondary hyperparathyroidism, and increase the risks of falls and vertebral and hip fractures [5]. Vitamin D deficiency is also associated with an increased risk of cardio metabolic disorders, infectious diseases and mortality, particularly in HIV-infected patients [6]. The risk factors of hypovitaminosis D in HIV infected patients are: a precarious social situation, high alcohol intake, coinfection with viral hepatitis, and/or a renal hydroxylation defect [7]. The impact of antiretroviral therapy (ARV) has also been raised, but the role of antiretroviral therapy on bone catabolism remains controversial [8-11].

While there is growing evidence that certain antiretroviral drugs affect vitamin D metabolism by increasing 25 OH vitamin D3 catabolism for efavirenz, or inhibiting renal hydroxylation of vitamin D for protease inhibitors (PIs), for example, all the potential mechanisms and their relevance remains unclear. On the other hand, it has been shown that inflammation markers (such as interleukin-6 (IL6) and highly-sensitive C-Reactive Protein (hsCRP)) correlated significantly with morbidity in patients on ARV [12,13]. It could thus be speculated that the different levels of residual inflammation on ARV could contribute to hypovitaminosis D and aggravate bone catabolism in HIV infected patients.

## Methods

Since no direct studies have been conducted yet, we carried out a cross-sectional study in an unselected cohort of 263 HIV infected outpatients consulting during Spring (March-June) 2010. This research was carried out in compliance with the Declaration of Helsinki. All of the patients included provided written informed consent to specifically participate to this study. According to current French research guidelines, no additional and specific ethical approval was needed since blood sampling was performed as part of the usual care in a single center without additional visit or sampling. All of the patients underwent a clinical examination, in particular to determine body mass index, Karnofsky’s index and to screen for lipodystrophy, and data on their medical history, food habits, sun exposure and addictions were collected. In particular, the immunological and virological efficacy and the type of past and present antiretroviral therapy were collected. In addition, the following data were systematically collected for each patient via a standardized questionnaire: present or past addiction, present and past personal and family history of bone fractures, endocrine disorders, chronic inflammatory diseases (enterocolopathy, inflammatory arthritis, cirrhosis, asthma and chronic obstructive pulmonary disease), treatments (in particular systemic glucocorticoids, and hormone therapy), functional status (history of falls during the previous year), age at menopause and amenorrhea for women, and orchidectomy for men. Sun exposure (expressed as minutes per day) was evaluated by the patients themselves. The daily intake of calcium was assessed using the questionnaire recommended by the French Research and Information Group on Osteoporosis (GRIO) [14]. Daily intake of alcohol was considered “excessive” when above 20 g per day in women and 30 g per day in men.

Fasting blood samples were taken from patients as part of standard care for immunological, virological, inflammation, endocrine and bone markers evaluations. Serum 25 OH vitamin D3 levels were measured by High Performance Liquid Chromatography (HPLC) coupled with fluorometry. Plasma total calcium, phosphorus, albumin and thyroid stimulating hormone (TSH) were quantified on a Vista analyzer (Siemens Healthcare Diagnostics, Erlangen, Germany) with dedicated reagents. A severe deficiency in 25 OH vitamin D3 was defined as a blood level below 10 ng/ml [25 nmol/l], by contrast with moderate deficiency (between 10 and 30 ng/ml) and normal levels (above 30 ng/ml – 75 nmol/l). Ionized calcium was measured on an ABL 800 analyzer (Radiometer, Bronshoj, Denmark). Serum PTH (Parathyroid hormon) was quantified on an Immulite 2500 analyzer (Siemens Healthcare Diagnostics, Erlangern, Germany) with dedicated reagents. Serum C-telopeptides X (CTX) were quantified using an ELISA kit (IDS Immunodiagnostic systems, France). The normal serum CTX value was defined according to sex and age (normal range in men: 0.115 -0.748 ng/ml, in pre-menopausal women: 0.112-0.738 ng/ml, and in menopausal women: 0.142 - 1.351 ng/ml). Inflammation was assessed by the measurement of hsCRP (immunonephelemetry on a VISTA analyzer, Siemens Healthcare Diagnostics, Erlangen, Germany) and IL6 (using the ultra sensitive ELISA kit, R&D System, Minneapolis, Minn, US).

Patients with severe deficiency were compared with those with moderate deficiency and those with normal levels. Categorical variables were compared using Fisher’s exact and chi-square tests, and continuous variables using the analysis of variance or the Mann–Whitney test when appropriate. Correlations between 25-OHD3 vitamin D levels and bone and inflammation markers were also analyzed by linear regression. In the second step, the patients with severe deficiency were compared with the others. A backward step-by-step logistic regression was then conducted for multivariate analysis of the factors associated with severe 25 OH vitamin D3 deficiency (dependent variables), including all of the factors associated in univariate analysis with a *p* value of less than 0.20. All of the tests were two-sided and a final p-value of less than 0.05 was considered statistically significant. All statistical analyses were performed using StatView® version 5.0.

## Results

The median age of the patients included was 47.7 years (range 20–72 years), and 188 (71.5%) were male. BMI was lower than 19 kg/m^2^ in 27 patients (10.2%) and higher than 25 kg/m^2^ in 83 patients (31.4%). Among the 75 women included, 33 (44%) were post-menopausal, and four were not menopausal, but suffering from amenorrhea. Twenty patients (7.6%) had a family history of hip fracture and 104 (39.5%) patients had a personal history of fracture: 15 had fragility fracture(s) (6.5%), and 82 had traumatic fracture(s) (35.7%) (undetermined in 7 patients). Long-term corticosteroid treatment was ongoing in 22 (8%) patients. Fifty-three patients were suffering from a chronic disease, including two cases of inflammatory rheumatic diseases, two cases of enterocolopathy, 23 cases of liver cirrhosis, and 24 cases of chronic bronchitis. Mean daily calcium intake was 760 (+/− 356) mg per day, and 149 (57%) patients had calcium intakes below 800 mg per day. Excessive alcohol consumption was reported in 86 (32%) cases.

The main current and past characteristics of HIV infection and antiretroviral therapy are presented in Table 1. The time since the HIV diagnosis was 13 years (+/− 8 years). Patients had very often been followed for several years and nearly one third of them (33.1%) had experienced AIDS defining illness. Most of them (91.6%) were on antiretroviral therapy, with a significant immune response, since the mean CD4 level at inclusion was 551 +/− 271/mm^3^, with a mean CD4 nadir of 198 +/− 190/mm^3^, and 207 (78.7%) patients had an undetectable viral load (<50 copies/ml).

**Table 1:** HIV-related characteristics of the 263 patients included

**Time elapsed since HIV diagnosis (years), mean +/− SD**	13 +/− 8
**CDC stage A, n (%)**	101 (38.4)
**CDC stage B, n (%)**	75 (28.5)
**CDC stage C, n (%)**	87 (33.1)
**Karnofsky’s index %, mean +/− SD**	92 +/− 14
**Time spent on NRTI (months), mean +/− SD**	95 +/− 71
**Time spent on tenofovir (months), mean +/− SD**	25 +/− 27
**Time spent on NNRTI (months), mean +/− SD**	40 +/− 47
**Time spent on PI (months), mean +/− SD**	44 +/− 47
**Without current antiretroviral therapy, n (%)**	22 (8.4)
**On HAART including NTI, n (%)**	210 (80.5)
**On HAART including tenofovir, n (%)**	164 (62.8)
**On HAART including NNTI, n (%)**	92 (35.4)
**On HAART including PI, n (%)**	151 (57.9)
**On HAART including II, n (%)**	21 (8.0)
**CD4 nadir (/mm3), mean +/− SD**	198 +/− 190
**Time spent with undetectable HIV viral load (months), mean +/− SD**	58 +/− 45
**Current CD4 (/mm3), mean +/− SD**	551 +/− 271
**Current CD8 (/mm3), mean +/− SD**	810 +/− 420
**Current CD4/CD8 ratio, mean +/− SD**	0.80 +/− 0.49
**Current undetectable HIV viral load, n (%)**	207 (78.7%)
**Current HIV viral load (when detectable), log**_**10**_**copies/ml +/− SD**	3.1 +/− 1.2

Ninety-five (36%) patients had SHD (Table 2). In univariate analysis, age, ethnicity, functional status (Karnofsky’s index and history of falls), daily sun exposure, tobacco use and hepatitis C coinfection were associated with SHD. The estimated duration of the HIV infection was also associated with hypovitaminosis D (p = 0.0008), whereas the HIV CDC stage, current and nadir CD4, HIV viral load, and time spent on the current ARV (by specific drug or overall), and the duration with undetectable HIV viral load were not.

**Table 2:** Associations between vitamin D status and clinical and therapeutic characteristics (univariate analysis) – only associated factors with a p value < 0.20 are shown in this table

		**All patients**	**Severe 25-(OH) vitamin D3 deficiency (<10ng/ml)**	**Moderate 25-(OH) vitamin D3 deficiency (10–30 ng/ml)**	**25-(OH) Vitamin D3 >30 ng/ml**	**p value**
		**N = 263**	**N = 95 (36%)**	**N = 135 (51%)**	**N = 33 (13%)**	
Sex	M, n (%)	188 (72%)	75 (79%)	89 (66%)	24 (69%)	0.10
	F, n (%)	75 (28%)	20 (21%)	46 (34%)	9 (31%)	
Age (years) mean +/− SD		48 +/− 10	49 +/−9	46 +/−10	51 +/−12	0.03
Ethnicity	non-African origin, n (%)	230 (87%)	87 (92%)	112 (83%)	31 (94%)	0.07
	African origin, n (%)	33 (13%)	8 (8%)	23 (17%)	2 (6%)	
Past history of falls, n (%)		120 (46%)	53 (57%)	55 (41%)	12 (36%)	0.03
Co-infections	Positive HBs antigenemia	14 (5.3%)	8 (8%)	6 (4%)	0 (0%)	0.14
	Positive HCV serology, n (%)	57 (22%)	33 (35%)	18 (14%)	6 (18%)	0.12
Past smokers, n (%)		52 (20%)	20 (21%)	25 (19%)	7 (21%)	0.003
Active smokers, n (%)		120 (46%)	55 (59%)	50 (37%)	14 (45%)	0.003
Sun exposure (min),mean +/− SD		106 +/− 114	93 +/− 108	102 +/− 111	157 +/− 132	0.02
Length of HIV infection (yrs), mean +/−SD		13 +/− 8	16 +/−8	12 +/−7	12 +/−8	0.0008
NRTI exposure (months), mean +/− SD		95 +/− 71	108 +/−70	89 +/−69	84 +/−73	0.08
Lipodystrophy, n (%)		65 (25%)	29 (31%)	31 (23%)	5 (15%)	0.005
Karnofsky’s Index (%), mean +/− SD		92 +/− 14	89 +/−17	92 +/−17	97+/−7	0.008

*M*: male, *F*: female, *NRTI*: nucleoside reverse transciptase inhibitor, *HBV*: hepatitis B infection, *HCV*: hepatitis C infection.

**Figure 1:**
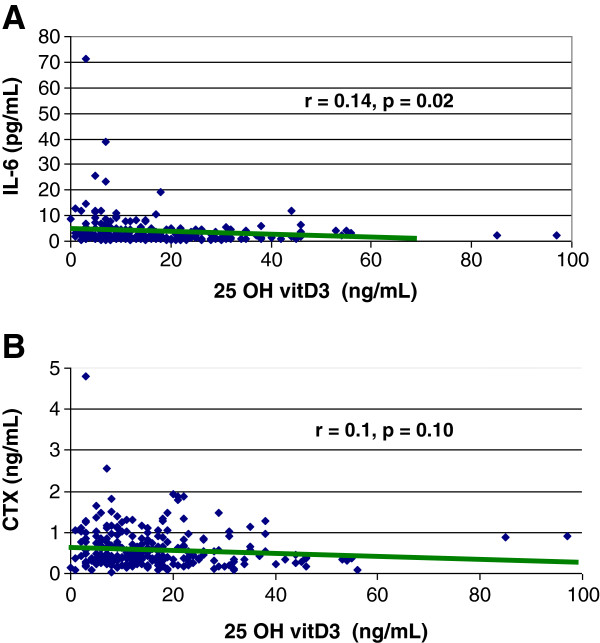
A and B: correlations between 25OH vitamin D3 levels and IL-6 (1A) or C-telopeptide X (1B).

A significant and positive association was also found between SHD and IL6 (p = 0.002), hsCRP (p = 0.12), increased serum C-Telopeptides X (CTX) (p = 0.05) and PTH (p < 0.0001) levels (Table 3). In univariate linear regression analysis, 25 OH vitamin D levels were found to correlate negatively with IL-6 levels (r = 0.14, p = 0.02, Figure 1A) and also tended to correlate negatively with serum CTX (r = 0.1, p = 0.10, Figure 1B), but not with hsCRP.

**Table 3:** Associations between vitamin D status and bone and inflammation biomarkers (univariate analysis)

		**All patients**	**Severe 25-(OH) vitamin D3 deficiency (<10ng/ml)**	**moderate 25-(OH) vitamin D3 deficiency (10–30 ng/ml)**	**25-(OH) Vitamin D3 >30 ng/ml**	**p value**
		**N = 263**	**N = 95 (36%)**	**N = 135 (51%)**	**N = 33 (13%)**	
Bone metabolism parameters	Ionized calcium <1.12 mmols/l, n (%)	9 (3%)	3 (3%)	3 (2%)	3 (9%)	0.001
	CTX (ng/mL), median (interquartile range)	0.45 (0.30-0.77)	0.49 (0.31-0.92)	0.45 (0.31-0.70)	0.33 (0.22-0.60)	0.05
	High serum CTX, n (%)	56 (21%)	29 (32%)	24 (18%)	3 (9%)	0.01
	Serum intact parathyroid hormone > 65 ng/l, n (%)	62 (24%)	31 (34%)	29 (21%)	2 (6%)	<0.0001
inflammation parameters	hsCRP (mg/l), median (interquartile range)	1.7 (0.7-4.0)	2.3 (1.2-5.3)	1.5 (0.9-2.7)	1.4 (0.8-4.2)	0.12
	IL6 (pg/ml), median (interquartile range)	1.8 (1.0-3.6)	2.0 (0.7-6.3)	1.7 (0.6-3.6)	1.6 (1.0-3.8)	0.002

*hsCRP*: highly-sensitive C-Reactive Protein; *IL6*: Interleukin 6; High serum *CTX* = C Telopeptide X > 0.748 ng/ml (men), 0.738 ng/ml (pre-menopausal women), and 1.351 ng/ml (menopausal women).

In multivariate analysis (Table 4), SHD deficiency remained significantly correlated with increased IL-6, but also with serum CTX levels above the normal value, lower mean daily exposure to the sun, current or past smoking, hepatitis C coinfection, and functional status. CTX levels expressed as quantitative values did not modify these results and remained significantly associated with SHD (OR +1 ng/ml, 1.96, 95% CI: 1.10-3.50, p = 0.02).

**Table 4:** Factors associated with severe hypovitaminosis D (< 10 ng/L) in multivariate analysis (final model - logistic regression)

		**Odds-ratio**	**95% Confidence Interval**	**p value**
Sun exposure (/+1 hour)		0.84	0.74-0.94	0.03
Past history of falls (yes vs. no)		1.80	1.00-3.27	0.05
Past smokers (yes vs. no)		2.74	1.17-6.43	0.02
Active smokers (yes vs. no)		2.63	1.27-5.45	0.009
Co-infections	Positive HCV serology (positive vs. negative)	1.87	0.92-3.85	0.09
	Positive anti-HBc and negative anti-HBs (yes vs. no)	2.76	1.17-6.54	0.005
Biological markers	IL-6 (+1 pg/mL)	1.13	1.02-1.25	0.02
	High serum CTX (yes vs. no)	2.44	1.23-4.81	0.01

*HCV*: Hepatitis C Virus; *HBV* Hepatitis B Virus; *hsCRP*: highly-sensitive C-Reactive Protein; *IL6*: Interleukin 6; High serum *CTX* = C Telopeptide X > 0.748 ng/ml (men), 0.738 ng/ml (pre-menopausal women), and 1.351 ng/ml (menopausal women).

## Discussion

In keeping with previous studies [1], vitamin D deficiency was highly prevalent in HIV-infected patients (close to 90% in our study cohort), with a prevalence of SHD ranging between 10 and 50% according to the places and the seasons [1,8,15-17]. However, the association with antiretroviral therapy appeared limited in our study, despite a significant number of patients on efavirenz, PIs and/or tenofovir. The first explanation could be that, by contrast with most previous studies, we also adjusted for the season (since blood samples were exclusively collected in spring 2010, at a time when vitamin D is synthesized). Sun exposure was found to correlate positively with vitamin D levels, and each additional one hour per day of exposure was associated with a significant reduction in the OR of SHD (0.84), which is consistent with previous data in non-HIV individuals.

Another explanation is suggested by the highly significant association between vitamin D levels and inflammation parameters we observed, which to date has never been reported. It can thus be suggested that HIV therapy could have an ambiguous impact on vitamin D levels. By reducing HIV viral replication and inflammation, ARV is likely to have a positive impact on vitamin D levels, which is counterbalanced by the potential direct negative effect of some drugs. We did not observe a statistically significant direct correlation between HIV viral load and inflammation biomarkers (data not shown). This association with inflammation, which has also been recently observed in non HIV infected patients [18-20], could also partly explain the increased risk of cardio-metabolic disorders associated with hypovitaminosis D, since hsCRP and IL6 have also been shown to be predictors of cardio-vascular and HIV disease progression and mortality [12,13,16].

Another result of interest is the association between vitamin D levels, inflammation and bone catabolism (in particular bone turnover markers CTX and PTH). CTX corresponds to the serum fraction of collagen called C telopeptid X, which is associated with osteoclast bone resorption. By contrast with the results from other study in HIV-infected patients, in our study, ionized calcium in the serum stayed within the normal range despite the vitamin D deficiency, suggesting an adaptive response to parathyroid hormone [1]. Even though we found a statistically significant association between SHD and inflammation and between SHD and increased bone catabolism, but not between inflammation and bone catabolism, the respective weights of hypovitaminosis D and inflammation in the increase in bone catabolism cannot be definitely and clearly established.

In addition, an association between SHD and a history of falls, which has never been reported in HIV-infected patients, was also observed in the present study. This proportion of patients with a history of falls is quite high, considering the median age of our study population. It may be partially due to the other characteristics of the patients included, all of whom were followed in a day-care hospital unit, had been infected for several years and had often experienced disease progression. Nevertheless, hypovitaminosis D has been shown to be associated with impaired muscle function in elderly people [21], and vitamin D supplementation led to improved muscle function and a decreased risk of falls in this population.

Last, we also observed a positive association between SHD and a history of viral hepatitis (positive HCV serology and “non-cured” HBV infection - i.e. positive HBc without anti-HBs antibodies), but we did not observe an association with HCV RNA or HBV DNA. The association with HCV seropositivity was previously reported in a recent study [15]. These HCV and HBV serological profiles can reflect a wide range of different realities. However, considering the number of our patients with known cirrhosis and/or with past or present chronic hepatitis viral replication, it could be argued that this relationship may be partially related to disorders in the hepatic hydroxylation of vitamin D associated with liver fibrosis [22]. Vitamin D deficiency was also found to be significantly associated with active or past smoking, as previously observed [23].

However, this study may suffer from several limitations, in part due to its cross-sectional design, since no repetitive measurements of vitamin D levels and inflammation parameters were performed, and thus no causal relationships can be determined. There was also no control group without HIV in order to compare the prevalence of hypovitaminosis D. In addition, the patients included may not have been representative, since they were followed in a French day-care hospital unit. On the other hand, a high proportion of these patients were exposed to HAART for a long period of time, which should strengthen the analysis of the association between hypovitaminosis D and HAART.

## Conclusions

Considering the high prevalence of SHD in our study and its association with increased inflammation markers, on the one hand, and with a history of falls and increased bone catabolism on the other, the results of our study may have three main implications. First, we believe that vitamin D levels have to be systematically and regularly assessed in HIV infected patients. Second, it may be of interest to take into account not only the type of antiretroviral therapy but also the residual inflammation on HAART in order to assess functional and fracture risks of HIV infected patients. Third, it is likely that there is an interest in supplementing hypovitaminosis D, at least because it is associated with falls and increased bone catabolism (as we observed) and thus fractures (as previously reported). However, whether increased bone catabolism is a process mainly driven by hypovitaminosis D rather than by residual inflammation is yet to be established, as is the best strategy for hypovitaminosis D screening and supplementation. It is now necessary to conduct longitudinal studies to assess not only the relationship between vitamin D levels and inflammation parameters, but also their potential clinical consequences, as well as the potential benefit of vitamin D supplementation.

## Competing interests

All the authors read and approved the final manuscript and have no conflict of interest with regard to the present article.

## Authors’ contributions

TA, CP, JFM, and LP conceived the study, and participated in its design and coordination and helped to draft the manuscript. SM, PO, PC enrolled the patients and helped to draft the manuscript. SE, JCD, LD carried out the assays and helped to draft the manuscript. TA, SM and LP performed the statistical analysis. All authors read and approved the final manuscript.

## Pre-publication history

The pre-publication history for this paper can be accessed here:

http://www.biomedcentral.com/1471-2334/13/7/prepub

## References

[B1] Rodriguez M Daniels B Gunawardene S Robbins GK High frequency of vitamin D deficiency in ambulatory HIV-Positive patients AIDS Res Hum Retroviruses 2009 25 9 14 10.1089/aid.2008.0183 19108690

[B2] Blanchard P Masterclass: HIV-infection and osteopathy Int J Osteop Med 2009 12 115 120 10.1016/j.ijosm.2009.04.001

[B3] Vescini F Cozzi-Lepri A Borderi M Re MC Maggiolo F De Luca A Prevalence of hypovitaminosis D and factors associated with vitamin D deficiency and morbidity among HIV-infected patients enrolled in a large Italian cohort J Acquir Immune Defic Syndr 2011 58 163 172 10.1097/QAI.0b013e31822e57e9 21826011

[B4] Viard JP Souberbielle JC Kirk O Reekie J Knysz B Losso M Vitamin D and clinical disease progression in HIV infection: results from the EuroSIDA study AIDS 2011 25 1305 1315 10.1097/QAD.0b013e328347f6f7 21522006

[B5] Bischoff-Ferrari HA Willett WC Orav EJ Lips P Meunier PJ Lyons RA A pooled analysis of vitamin D dose requirements for fracture prevention N Engl J Med 2012 367 40 49 10.1056/NEJMoa1109617 22762317

[B6] Parker J Hashmi O Dutton D Mavrodaris A Stranges S Kandala NB Levels of vitamin D and cardiometabolic disorders: systematic review and meta-analysis Maturitas 2010 65 225 236 10.1016/j.maturitas.2009.12.013 20031348

[B7] Overton ET Yin MT The rapidly evolving research on vitamin D among HIV-infected populations Curr Infect Dis Rep 2011 13 83 93 10.1007/s11908-010-0144-x 21308459

[B8] Van Den Bout-Van Den Beukel CJ Fievez L Michels M Sweep FC Hermus AR Bosch ME Vitamin D deficiency among HIV type 1-infected individuals in the Netherlands: effects of antiretroviral therapy AIDS Res Hum Retroviruses 2008 24 1375 1382 10.1089/aid.2008.0058 18928396

[B9] Libois A Clumeck N Kabeya K Gerard M De Wit S Poll B Risk factors of osteopenia in HIV-infected women: no role of antiretroviral therapy Maturitas 2010 65 51 54 10.1016/j.maturitas.2009.10.009 19939594

[B10] Piso RJ Rothen M Rothen JP Stahl M Markers of bone turnover are elevated in patients with antiretroviral treatment independent of the substance used J Acquir Immune Defic Syndr 2011 56 320 324 10.1097/QAI.0b013e31820cf010 21350365

[B11] McComsey GA Kitch D Daar ES Tierney C Jahed NC Tebas P Bone mineral density and fractures in antiretroviral-naive persons randomized to receive abacavir-lamivudine or tenofovir disoproxil fumarate-emtricitabine along with efavirenz or atazanavir-ritonavir: aids clinical trials group A5224s, a substudy of ACTG A5202 J Infect Dis 2011 203 1791 1801 10.1093/infdis/jir188 21606537 PMC3100514

[B12] Lau B Sharrett AR Kingsley LA Post W Palella FJ Visscher B Gange SJ C-reactive protein is a marker for human immunodeficiency virus disease progression Arch Intern Med 2006 166 64 70 10.1001/archinte.166.1.64 16401812

[B13] Kuller LH Tracy R Belloso W De Wit S Drummond F Lane HC Inflammatory and coagulation biomarkers and mortality in patients with HIV infection PLoS Med 2008 5 e203 10.1371/journal.pmed.0050203 18942885 PMC2570418

[B14] Fardellone P Sebert JL Bouraya M Bonidan O Leclercq G Doutrellot C [Evaluation of the calcium content of diet by frequential self-questionnaire] Rev Rhum Mal Osteoartic 1991 58 99 103 2042014

[B15] Mueller NJ Fux CA Ledergerber B Elzi L Schmid P Dang T High prevalence of severe vitamin D deficiency in combined antiretroviral therapy-naive and successfully treated Swiss HIV patients AIDS 2010 24 1127 1134 10.1097/QAD.0b013e328337b161 20168200

[B16] Calmy A Gayet-Ageron A Montecucco F Nguyen A Mach F Burger F HIV increases markers of cardiovascular risk: results from a randomized, treatment interruption trial AIDS 2009 23 929 939 10.1097/QAD.0b013e32832995fa 19425222

[B17] Dao CN Patel P Overton ET Rhame F Pals SL Johnson C Low vitamin D among HIV-infected adults: prevalence of and risk factors for low vitamin D Levels in a cohort of HIV-infected adults and comparison to prevalence among adults in the US general population Clin Infect Dis 2011 52 396 405 10.1093/cid/ciq158 21217186

[B18] Bucharles S Barberato SH Stinghen AE Gruber B Meister H Mehl A Hypovitaminosis D is associated with systemic inflammation and concentric myocardial geometric pattern in hemodialysis patients with low iPTH levels Nephron Clin Pract 2011 118 c384 c391 10.1159/000323664 21325871

[B19] Bellia A Garcovich C D'Adamo M Lombardo M Tesauro M Donadel G Serum 25-hydroxyvitamin D levels are inversely associated with systemic inflammation in severe obese subjects Intern Emerg Med 2011 Epub ahead of print 10.1007/s11739-011-0559-x 21437585

[B20] Guillot X Semerano L Saidenberg-Kermanac’h N Falgarone G Boissier MC Vitamin D and inflammation Joint Bone Spine 2010 77 552 557 10.1016/j.jbspin.2010.09.018 21067953

[B21] Bischoff-Ferrari HA Dawson-Hughes B Willett WC Staehelin HB Bazemore MG Zee RY Wong JB Effect of Vitamin D on falls: a meta-analysis JAMA 2004 291 1999 2006 10.1001/jama.291.16.1999 15113819

[B22] George J Ganesh HK Acharya S Bandgar TR Shivane V Karvat A Bone mineral density and disorders of mineral metabolism in chronic liver disease World J Gastroenterol 2009 15 3516 3522 10.3748/wjg.15.3516 19630107 PMC2715978

[B23] Wasserman P Rubin DS Highly prevalent vitamin D deficiency and insufficiency in an urban cohort of HIV-infected men under care AIDS Patient Care STDS 2010 24 223 227 10.1089/apc.2009.0241 20377437

